# Environmental Health Organisations against Tobacco

**DOI:** 10.3390/ijerph6041456

**Published:** 2009-04-15

**Authors:** Maurice Mulcahy, David S. Evans, Blaithin Lahiffe, Deirdre Goggin, Colm Smyth, Gerard Hastings, Miriam Byrne

**Affiliations:** 1 Department of Environmental Health, Health Service Executive West, Galway, Republic of Ireland; E-Mail: blaithin.lahiffe@hse.ie; 2 Department of Public Health, Health Service Executive West, Galway, Republic of Ireland; E-Mails: david.evans@hse.ie (D.S.E.); deirdre.goggin@hse.ie (D.G.); 3 Department of Environmental Health, Dublin City Council, Dublin, Republic of Ireland; E-Mail: colm.smyth@dublincity.ie; 4 Cancer Research UK Centre for Tobacco Control Research, University of Stirling and the Open University, U.K.; E-Mail: Gerard.hastings@stir.ac.uk; 5 Department of Experimental Physics at National University of Ireland, Galway, Republic of Ireland; E-Mail: miriam.byrne@nuigalway.ie

**Keywords:** Anti tobacco policy making, enforcement, environmental health, health professionals, tobacco control

## Abstract

Implementing the World Health Organisation (WHO) Framework Convention on Tobacco Control (FCTC) relies heavily on enforcement. Little is known of the way different enforcement agencies operate, prioritise or network. A questionnaire was sent to representatives of the International Federation of Environmental Health (IFEH) in 36 countries. Tobacco control was given low priority. Almost two thirds did not have any tobacco control policy. A third reported their organisation had worked with other agencies on tobacco control. Obstacles to addressing tobacco control included a lack of resources (61%) and absence of a coherent strategy (39%).

## 1. Introduction

The World Health Organisation (WHO) Framework Convention on Tobacco Control (FCTC) [1] was the world’s first public health treaty. It was developed in response to the globalisation of the tobacco epidemic and aims to protect present and future generations from tobacco consumption and exposure to tobacco smoke. It commits countries that ratify the treaty to implement a range of evidence based tobacco control measures addressing both the demand and supply of tobacco. As of July 2008, 168 nations have signed the treaty, with ratification by 157 [2]. The challenge now facing these countries is the effective translation of the treaty into national legislation and effective enforcement and educational programmes so that high compliance rates result.

As this research focuses particularly on the role of enforcement it is important to note that barriers to the success of enforcement programmes include the lack of awareness of the tobacco problem amongst the public and policy makers and the continuing opposition of the tobacco industry and its affiliates [3,4]. Conversely, effective implementation will require comprehensive tobacco control programmes encompassing prevention, protection, cessation, and harm reduction with dedicated national agencies free from all tobacco industry influence [5]. It is also recognised that the enforcement infrastructures required to deliver tobacco free societies, should have the necessary technical expertise, information systems, skilled management, public support, strong political leadership, the necessary legislation and increasing resources at the country level [6]. Furthermore, they should be strategic with effective planning and co-ordination, have well trained inspectors, clear lines of authority, be capable of dealing with outright defiance and contain a public education programme [7].

Environmental Health Practitioners (EHP’s) “strive to promote health and quality of life by preventing or controlling those diseases or deaths that result from interactions between people and their environment” [8]. Moreover, EHP’s in many countries have the responsibility for the enforcement of smoking restrictions and controls on sales of tobacco to minors. The phrase Environmental Health Practitioner (EHP) is an umbrella term used here to describe the organizations and members affiliated to the International Federation of Environmental Health (IFEH). It includes those working as Environmental Health Officers, Public Health Inspectors, Public Health Officers and Health Inspectors.

The role of health professionals such as EHP’s has been recognised by the WHO in the FCTC [1]. It has also developed a Code of Practice for Health Professional Organisations [9]. This Code emphasises the role of health professionals in tobacco control and has identified 14 steps needed for them to contribute to global efforts to reduce the negative impact of tobacco use.

As regulators, it would appear that EHP’s are in the most crucial of positions to curb the tobacco epidemic; however, their role has never been assessed globally. It is not known if IFEH organisations are actively pursuing the complimentary objectives of the Code of Practice and the FCTC by way of their policies and practices. As tobacco legislation has been partly differentiated from other public protection legislation because of tobacco industry influence [3], it is important to establish if such organisations refrain from accepting tobacco industry support and have policies to ensure they were ‘fire walled’ against such tobacco industry influence.

It was against this background that the study was undertaken. It assessed the role of EHP’s in tobacco control, identified obstacles to effective tobacco control and benchmarked existing tobacco control policies against the WHO code of practice for professional organisations.

## 2. Methods

In any particular country, there may be a number of different agencies involved in the enforcement of tobacco control regulations. To provide an overview for each country, it was therefore decided to seek feedback from national representatives of the International Federation of Environmental Health (IFEH). The IFEH aims to provide a focal point for national organisations of practitioners of environmental health, whether in state, local government, or private employment, whose concern is the care of the environment in the interests of the public [10]. The IFEH represents national organisations that are involved in Environmental Health in 37 countries (36 at the time of the survey) and is the only body with such global reach in terms of its EHP membership. The IFEH network of organisations is therefore well placed to give information on the enforcement of tobacco controls by EHP’s in their individual countries.

The IFEH national contact person from the 36 countries that were full members of the IFEH at the time of the study were contacted by e-mail and asked to participate in an online survey to elicit information about tobacco control issues in their country. The national contacts were senior representatives of their own national organisations, such as President/CEO (38%), and honorary secretary (26%). Two reminder e-mails one week and two weeks after the initial e-mail were sent, followed by one telephone call one week after the second e-mail (to ensure non responders were the appropriate respondents to complete the survey and to encourage participation). In countries where more than one national representative completed the survey (four cases), only the responses from the first respondent to return the survey were included in the study. From their knowledge of their members’ activities, respondents were asked to complete the online questionnaire to reflect the current position in their country. This, combined with the collection of factual information (as opposed to subjective opinion) helped to minimise bias from individual responses. This approach of contacting well placed individuals to give information on the implementation of tobacco control policies at country level has also been utilised by Jossens and Raw [11].

Based on a review of the WHO Code of Practice for Professional Organisations [9] and the Framework Convention for Tobacco Control [1], the questionnaire sought to establish the areas covered by tobacco legislation in their country (based on categories used by the American Cancer Society to describe national tobacco legislation [12]), the level of involvement their organisation had in developing the policy, and the level of enforcement by EHP’s. They were also given six environmental health issues (housing, sanitation, drinking water, tobacco control, food hygiene, and communicable disease) and asked to rank them in terms of priority for their IFEH organisation. In addition, the level of inter-agency working to develop joint strategies and policies in tobacco control was assessed. Respondents who stated that their IFEH organisation had a policy on tobacco control were given a list of 23 items and asked whether each item was included in their policy. These items were based on the Code of Practice for Professional Organisations [9] and a score out of 23 was calculated which was then weighted to give a score out of 100 for each respondent to assess performance against the Code of Practice [9]. Six obstacles to addressing tobacco control issues were presented to respondents who were asked whether they were obstacles to their IFEH organisation. These were based on obstacles identified by the WHO for health professionals [9]. Given the spread of second hand smoke (SHS) controls across the globe and the opportunity for EHPs to gather evidence of improvements in air quality and reductions in associated health risks [13–21] respondents were asked if there individual members had the skills to accurately measure SHS and if a guide to the measurement of SHS exposure would be of value.

Prior to its administration, the questionnaire was piloted on six environmental health officers (of varying seniority) employed by the Health Service Executive in the Republic of Ireland and feedback obtained in terms of structure, content, and layout. The questionnaire was administered using an online survey tool (Zoomerang) and data was analysed using SPSS^®^ v.15.

## 3. Results

### 3.1. Introduction

Responses to the survey of IFEH organisations were received from 19 out of 36 countries giving a 53% response rate (Table 1). Over half the responses (52%) were from European countries, 21% from Africa, 11% from Asia, and 16% from Canada, USA, and Australia. The majority of countries responding were high income ones, according to World Bank classification [22]. Of the remainder, two of the eight countries classified as low income responded (Kenya and Zimbabwe) and two of those classified as upper middle income responded (Lithuania and South Africa).

### 3.2. National Legislation and Role in its Enforcement

All respondents reported that their country had some national legislation covering tobacco control. Over two thirds of respondents (68%) stated that their IFEH organisation had little or no involvement in developing the legislation.

Table 2 (columns a and b) shows the areas covered by the legislation and the areas enforced by EHP’s. It can be seen that for all but one of the 15 legislation areas, over half of the respondents stated that their national legislation covered these areas. The mean number of areas covered for each country was 10.32 (sd = 4.73). The most predominant areas of legislation were packaging and labelling of tobacco products (95%), sales of tobacco to children under a specified age (84%), advertising in certain locations (74%), advertising in certain media (74%), and smoking in public buildings (74%). The areas that the fewest countries legislated for included counterfeit tobacco products (47%), brand stretching (53%), and free tobacco products (58%).

In terms of the enforcement of legislation by EHP’s, Table 2 (columns c and d) shows that the majority of respondents stated that nine of the 15 elements were enforced. The mean number of areas enforced for each country was 5.47 (sd = 3.70). The most areas enforced in any one country by EHP’s was fourteen and the least was none. The areas of legislation enforced by the greatest proportion of EHP’s were smoking in restaurants (92%), smoking in public buildings (86%), advertising in certain locations (79%) and sales of tobacco to children under a specified age (69%). The areas of legislation enforced by the lowest proportion of EHP’s were smuggling of tobacco products (no EHP enforcement reported), counterfeit tobacco products (22%), brand stretching (30%), and regulation on the content of tobacco products (31%).

### 3.3. Priority Given to Tobacco Control

**Figure 1.**
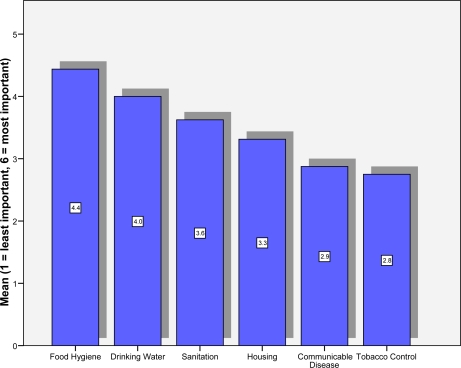
Mean Priority Score for Environmental Health Issues.

Figure 1 shows the mean ranking given to six environmental health issues (6 = most important, 1 = least important). It can be seen that issues prioritised as most important were food hygiene (mean = 4.4) and drinking water (mean = 4.0). Tobacco control was given the least important rating in terms of priority (mean = 2.8). Respondents who stated that their own IFEH organisation had a policy on tobacco control gave higher priority to tobacco control than those who did not have a policy (mean = 3.86 compared to 2.36). These differences were statistically significant (Independent T test, t = −2.077, df = 16, p = 0.054). There were no significant differences in the priority given to tobacco control for countries within the European Union and those outside the European Union (Mean = 3.23 compared to 2.63, Independent T Test, t = 0.735, df = 16, p = 0.473).

Table 3 shows the frequency that IFEH organisations highlight tobacco issues through publications and organised events and it can be seen that less than a quarter frequently highlight tobacco issues (16–21%). Almost half of respondents stated that tobacco issues were occasionally highlighted in environmental health publications (47%) and at conferences (42%). Approximately two thirds seldom or never highlighted tobacco issues at training days (69%) and environmental health representative body meetings (61%). Although only 40% of respondents felt that their members did not have the necessary skills to measure SHS, the majority (90%) felt a guide would be of value.

### 3.4. Working with Other Agencies

A third of respondents (33%) reported that their IFEH organisation had worked with other agencies in the last year to develop joint strategies or policies in tobacco control. In addition, a quarter (26%) stated their IFEH organisation had worked in the last year with other agencies to develop anti-tobacco campaigns.

### 3.5. Policy on Tobacco Control

Almost two thirds of respondents (63%) stated that their IFEH organization did not have a policy on tobacco control. Table 4 shows that for those that did have a policy (37%: 7 respondents), the areas covered by the greatest proportion of respondents were encouraging members to be tobacco free at its organisations events (86%), prohibiting the sale (86%) and promotion (86%) of tobacco products in their own organisations premises (86%), and supporting campaigns for tobacco free public places (86%).

In terms of ‘fire walling’ of organisations against tobacco industry influence, 57% stated that their policy refrained from accepting any kind of support form the tobacco industry and 57% encouraged their members to refrain from accepting such support. Likewise, 57% had a stated policy on commercial or other kinds of relationships with partners who have interests in the tobacco industry through a declaration of interest. The same four countries included ‘fire walling’ in their policy in terms of these three ‘fire-walling’ issues.

Four items were not reported to be covered by any respondent in their policy. These included assessing the tobacco consumption patterns of individual members through surveys, advising members to routinely ask clients about tobacco consumption and exposure to tobacco products, and advising individual members to routinely give advice to clients on smoking cessation.

**Figure 2.**
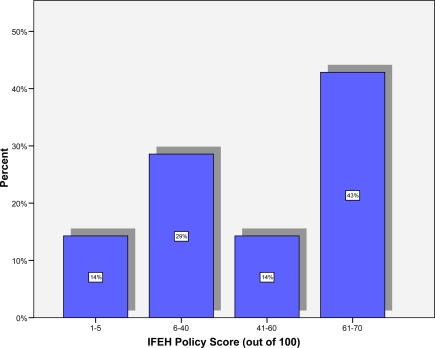
IFEH Policy Score (Based on [9]).

For the 7 countries that had a policy on tobacco control, Figure 2 provides an overall policy score out of 100, based on the Code of Practice for Professional Organisations [9] (weighted score of the 23 items shown in Table 4). It can be seen that 43% scored between 61 and 70 out of 100, while 43% scored 40 or less and 14% scored between 41 and 60. No IFEH policy scored over 70 out of 100. The mean score was 45.97 (median = 52.17, SD = 24.19).

### 3.6. Obstacles

**Figure 3.**
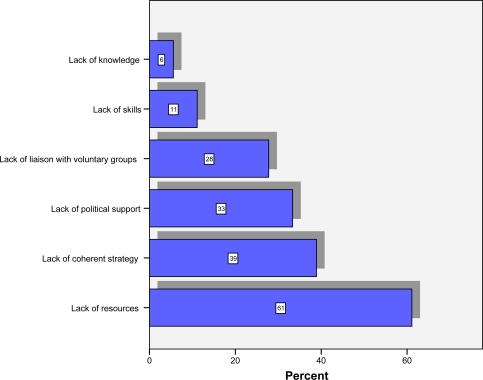
Obstacles to IFEH Organisation in Addressing Tobacco Control Issues.

Figure 3 shows that the main obstacles for IFEH organisations in terms of addressing tobacco control were lack of resources (61%), lack of coherent strategy (39%), lack of political support (33%), and a lack of liaison with voluntary groups (28%). Almost a third of respondents (33%) also stated other obstacles including priority being given to food hygiene (33%), no national legislation (17%), and interference from tobacco manufacturers (17%).

## 4. Discussion

From our survey of the member organisations of the IFEH, it is apparent that EHPs’ have an important role to play in enforcing tobacco control legislation and delivering on the FCTC [1]. Through IFEH representatives, the study provides an overview of current involvement by EHP’s in tobacco control across 19 countries. The IFEH does not represent all enforcement officers, nor all EHPs, nor all EHP organisations and so this must be noted as a limitation of this study. Many countries where tobacco consumption is a significant problem (e.g. China, Japan, Russia and Indonesia) do not have an IFEH organisation and also were not included in the study.

Given the absence of an international directory of tobacco control enforcement agencies, we chose to sample a profession involved in tobacco control, specifically the environmental health profession. In selecting a profession represented in a number of countries it was to be expected that there would be some variability in national arrangements for tobacco control including perhaps some respondents having little or no involvement in this field. This could be for several reasons including the absence of national objectives in tobacco control. Indeed when we matched non responders to the presence of national objectives (using MPOWER [23] policy data) 65% had no national tobacco objectives. This demonstrates the need for widespread adoption of national objectives and a comprehensive directory of agencies.

The study is also limited in that it does not permit statistical analysis on a country-by-country basis. In addition it is recognised that relying as it does on individual respondents from each EHP organisation introduces the potential for bias. However, using national representatives within IFEH (who it could be assumed through their role would be well informed of the situation in their own country) and obtaining a 53% response rate does provide a valuable insight into a range of issues surrounding tobacco control implementation worldwide.

If tobacco control programmes are to be successful, it is essential that comprehensive legislation is put in place and that it is adequately enforced. Whilst all 19 countries had national legislation, on average, only ten out of 15 areas were covered by the legislation and no country had legislation covering the full 15 areas. This is promising but does show scope for improvement. If the FCTC^1^ is to be fully implemented, individual countries will need to match their ratification of the Treaty with comprehensive legislation that is informed by relevant guidance.

Of the 15 key legislative areas required, the mean number provided for each country was 10.32 of which an average of 5.47 was enforced by EHPs. The most areas enforced in any one country by EHP’s were 14 and the least was zero. As these figures indicate potential gaps in the enforcement of tobacco legislation and weaknesses in enforcement infrastructures, it is advisable that States conduct comprehensive audits to ensure that the FCTC is being transposed carefully into national laws, that legal powers are duly delegated to EHPs /enforcement agencies or other officials and that active enforcement is resulting in high levels of compliance. This is essential as failure to enforce legislation may have extremely negative short term effects such as greater non-compliance with smoking restrictions [24], and increases in sales of tobacco to minors [25]. Widespread failure to enforce legislation over the longer term, may result in the worst predictions of smoking related harm [23] being realised.

The regulatory implications of pursuing the objectives of the FCTC [1] are many and diverse. Hence the FCTC [1] has implications for several organisations in the state sector (including, Health Authorities, Local Authorities, Police, Government Departments) and professions (including Environmental Health Officers, Trading Standards Officers, Laboratory Scientists, Customs Officials, State Solicitors, Health and Safety Inspectors and Officers of National Offices of Tobacco Control). To be effective, there is consequently a need for a multisectoral approach to enforcement, multi agency working and joint strategies/policies. Now, only a third of IFEH organisations are involved in multi agency working to develop joint strategies and policies in tobacco control. This would indicate that relevant States should develop multisectoral strategies to ensure comprehensive enforcement and as obliged to do so by the FCTC [1] this would be best arranged by a ‘focal point’ agency specialising in tobacco control. States should also not overlook the immense benefits of networking with agencies/professionals in other countries to transfer knowledge and best practice (a guiding principle of the FCTC [1]). International collaborations appear to have worked successfully in other areas of public protection. For example, in food safety, where there has been a sharing of enforcement best practices, the exchange of intelligence and the development of enforcement campaigns across national boundaries. The Food Law Enforcement Practitioners network (FLEP) is an informal grouping of European food law enforcement practitioners. The aims include acquaintance, exchange of information and cooperation between European colleagues in order to further develop mutual confidence and trust in the resolution of practical control problems [26]. The European Food Safety Authority (EFSA) [27]. Advisory Forum is on the other hand, a more formal arrangement. It connects EFSA with the national food safety authorities of all 27 EU Member States. Its members represent each national body responsible for risk assessment in the EU, with observers from Norway, Iceland, Switzerland and the European Commission. Through it, EFSA and the Member States can join forces in addressing European risk assessment and risk communications issues. The Forum also helps national authorities share information and co-ordinate activities between themselves. Whilst informal partnership approaches do exist for example in the monitoring of the FCTC [28] urgent consideration should be given to the establishment of official and regional tobacco control agencies, as already called for in Europe [5].

Along with a multi-agency approach, EHP’s involvement in enforcing legislation would be greatly enhanced if tobacco control were prioritised by member organisations of the IFEH. At the moment, the study indicates that this is not the case. Of the six environmental health issues presented to respondents, tobacco control was ranked the lowest in terms of priority. Although it is acknowledged that other environmental health issues are important, tobacco control should be given greater priority as it is the leading preventable cause of death worldwide, killing 5.4 million people per year [23]. Prioritisation of environmental health issues should be based on their burden in terms of death and disease. The lack of priority given to tobacco control is also highlighted by the fact that 63% of IFEH organisations did not have a policy on tobacco control. Moreover, those that had a policy only scored 46 on average out of 100 in terms of it addressing areas recommended in the Code of Practice of Professional Organisations [9]. Only the EHP organisations of four countries had policies that met with its recommendations on refraining from accepting support from the tobacco industry or engaging with partners with such interests.

It is disappointing that less than a quarter of organisations frequently highlighted tobacco issues by way of training events, conferences and publications because these activities assist individual members maintain professional competence in tobacco control. Specifically they can highlight enforcement difficulties, legal case law and aid compliance building by explaining the public health reasoning behind smoke free or other tobacco control provisions. If enforcement officers understand the particular significance of legislation they are then more able to convince businesses as to its benefits.

The major obstacles to effective tobacco control were lack of sufficient resources and coherent strategies. Our findings indicate that having a policy on tobacco control will increase the priority it is given and so this points to the need for all IFEH organisations to develop tobacco control policies, given the enormous negative health impact tobacco use has when compared to other environmental health issues.

The tobacco industry continues to attempt to influence other organisations. For example, in California, the tobacco industry spent $4,359,205 in 2005/6 on political contributions [29]. Tobacco industry campaign contributions have been found to influence the tobacco control policy making of state policy makers in USA [30]. The tobacco industry has also sought to undermine public health and create doubt on issues such as the control of SHS [31–34]. If IFEH organisations do not have systems to ‘firewall’ against this influence, then the tobacco industry may influence the way they and EHP’s work. Organisations need to adopt robust policies and governance to prevent the receipt of sponsorship monies or other commercial linkages and requirements for individual members to openly declare any related interests [9]. As tobacco control advocates contemplate areas of vulnerability to tobacco control influence [35] overlooking the potential for corruption of enforcement officials/agencies seems a gross oversight.

## 5. Conclusions

The FCTC [1] has enormous potential in terms of helping to prevent tobacco related harm and requires concerted action at both national and international levels. This is the first International survey of a professional grouping charged with enforcement responsibilities in tobacco control. It gives an important insight into the conflicting priorities that exist for this profession and indicates a potential vulnerability to tobacco industry influence; as a result of the widespread absence of appropriate ‘firewall’ policies From this research of Environmental Health Practitioner Organisations there is a need for priorities to be reassessed, for tobacco policies to be put in place and for leadership to be shown in working with other agencies so as to develop effective enforcement infrastructures. In so doing and as part of an overall package that includes adequate tax rises and cessation programmes there is the real potential to reverse the tobacco epidemic [23]. Surely, for those working in Environmental Health (particularly the IFEH) there is no other option but to pursue this worthy objective with vigour. We recommend that an international directory of tobacco control agencies and officials be compiled to facilitate both future networking and research.

## Figures and Tables

**Table 1.** Member Organisations of the IFEH by Country (respondents to questionnaire in bold).

***Country and income classification***	***Name of Organisation***
**Australia^h^**	Australian Institute of Environmental Health
**Austria^h^**	Verband der Osterreichischen Lebensmittelkontrolleure
Botswana ^um^	Botswana Environmental Health Officers Association
**Canada^h^**	Canadian Institute of Public Health Inspectors
Cyprus **^h^**	Association of Public Health Inspectors of Cyprus
**Denmark^h^**	FMK Denmark
**England, Wales & North Ireland^h^**	Chartered Institute of Environmental Health
**Finland^h^**	Finnish Communal Association of Environmental Health and Protection
**France^h^**	Association Nationale des Ingenieurs du Genie Sanitaire
Germany **^h^**	Bundesverband der Lebensmittelkontrolleure
Hong Kong **^h^**	Hong Kong Public Health Inspector's Association
**Republic of Ireland^h^**	Environmental Health Officers Association
Jamaica ^um^	Jamaican Association of Public Health Inspectors
**Kenya^l^**	Association of Public Health Officers - Kenya
Latvia ^um^	Latvian Association of Preventive Medicine
Liberia **^l^**	Liberia Association of Public Health Inspectors
**Lithuania**^um^	Lithuanian Union of Hygienists & Epidemiologists
Malaysia ^um^	Malaysian Association of Environmental Health
Malawi	Environmental Health Officers Association of Malawi
**Malta^h^**	Malta Environmental Health Officers Association
Mauritius ^um^	Health Inspectors Cadre, Mauritius
**Netherlands^h^**	College van Keurmeesters Netherlands
**New Zealand^h^**	New Zealand Institute of Environmental Health Inc,
Nigeria	Environmental Health Society of Nigeria (EHSoN)
Norway **^h^**	Forum for Miljø Og Helse
Rwanda **^l^**	Rwanda Association of Environmental Health
**Scotland^h^**	Royal Environmental Health Institute of Scotland
**Singapore^h^**	Society of Environmental Health, Singapore
**South Africa**^um^	South African Institute of Environmental Health
Sri Lanka	Public Health Inspectors Union of Sri Lanka
**Sweden^h^**	Association of Environmental Health Professionals (Sweden)
Tanzania **^l^**	Tanzanian Association of Health Inspectors (Chama cha Maafisa wa Afya Tanzania)
Uganda **^l^**	Environmental Health Workers Association of Uganda
**United States^h^**	National Environmental Health Association
Zambia **^l^**	Zambian Institute of Environmental Health
**Zimbabw**e **^l^**	Zimbabwe Association of Environmental Health Practitioners

**Annotations** h = high income country classification, um = upper middle income classification **,** l- low income classification. Source World Bank [22].

**Table 2.** Areas Covered by National Tobacco Legislation and EHP’s Enforcement.

*Areas of legislation*	*Areas covered by National Legislation (a) (b)*		*Areas Enforced by EHP’S (c) (d)*	
	Number of countries	% *	Number of countries	% *
Smoking in restaurants	13	69	12	92
Smoking in bars	11	58	10	91
Smoking in public transport	13	68	8	62
Smoking in public buildings	14	74	12	86
Sales of tobacco to children under a specified age	16	84	11	69
Advertising in certain locations	14	74	11	79
Advertising in certain media	14	74	6	43
Sponsorship	12	63	4	33
Brand stretching	10	53	3	30
Packaging and labelling of tobacco products	18	95	7	39
Single cigarette sales	12	63	7	58
Free tobacco products	11	58	6	55
Smuggling of tobacco products	13	68	0	0
Counterfeit tobacco products	9	47	2	22
Regulation on content of tobacco products	16	84	5	31
Summary statistics	Mean = 10.32, sd = 4.73, minimum = 2, maximum = 15,		Mean = 5.47, sd = 3.70, minimum = 0, maximum = 14,	

* Multiple Response, therefore percentages may not add up to 100%.

**Table 3.** Frequency Tobacco Issues Highlighted by IFEH Organisation.

**Frequency highlighted**	***Conferences***		***Environmental health publications***		***Training days***		***Environmental health representative body meetings***	
	**No.**	**%**	**No.**	**%**	**No.**	**%**	**No.**	**%**
Frequently	4	21	3	16	2	11	3	17
Occasionally	8	42	9	47	3	16	2	11
Seldom	4	21	3	16	6	32	6	33
Never	3	16	3	16	7	37	5	28
Don t know	0	0	1	5	1	5	2	11

**Table 4.** Areas covered by policy on tobacco control (based solely upon [9]).

***Areas covered***	***No.***	% *
Encourage members to be role models by not using tobacco	3	43
Assess the tobacco consumption patterns of members through surveys	0	0
Assess the tobacco consumption patterns of members by the introduction of appropriate policies	1	14
Make events run by the organisation tobacco free	4	57
Encourage members to be tobacco free at its organisations events	6	86
Include tobacco control on the agenda of relevant health related congresses	5	71
Advise members to routinely ask clients about tobacco consumption	0	0
Advise members to routinely ask clients about exposure to tobacco products	0	0
Advice members to routinely give advice to clients on smoking cessation	0	0
Influence health institutions to include tobacco control in their health professional curricula	2	29
Influence educational centres to include tobacco control in their health professional curricula	3	43
Actively participate in “No tobacco day” every May 31^st^	3	43
Refrain from accepting support, (financial or otherwise) from the tobacco industry	4	57
Encourage members to refrain from accepting any kind of support (financial or otherwise) from the tobacco industry	4	57
Ensure own organisation has a stated policy on any commercial or other kind of relationship with partners who have interests in the tobacco industry through a declaration of interest	4	57
Prohibit the sale of tobacco products in own organisations premises	6	86
Prohibit the promotion of tobacco products in own organisations premises	6	86
Actively support government in the process leading to the signature, ratification, and implementation of the WHO Framework Convention on Tobacco Control	4	57
Dedicate financial resources to tobacco control	3	43
Dedicate other resources to tobacco control	5	71
Dedicate resources to the implementation of the Code of Practice	1	14
Participate in tobacco control activities of health professional networks	4	57
Support campaigns for tobacco free public places	6	86

* Multiple Response, therefore percentages may not add up to 100%.
